# Supplementary material for: MetaRibo-Seq measures translation in microbiomes
Source: Nat Commun. 2020 Jun 29;11:3268. doi: 10.1038/s41467-020-17081-z (PMC7324362; doi:10.1038/s41467-020-17081-z)
Supplement: Supplementary file 10 — Supplementary Data 7 [file 41467_2020_17081_MOESM10_ESM.zip › File2/Confidence_High_Taxonomy/319464_out.krona.html]

Javascript must be enabled to view this page.

members
magnitude
magnitudeUnassigned
count
unassigned
taxon
rank

319464\_out

251

251
2
superkingdom

976
phylum
249

1
117743
class

200644
order
1

family
49546
1

1
1016
genus


SRS016541\_contig\_number\_contig-100\_109.106316
1
1017
species

248
class
200643

order
171549
248

family
2005519
1

genus
1348911
1

1

SRS064276\_contig\_number\_20053
species
1941478

2005525
family
202

202
genus
375288


SRS011302\_contig\_number\_25466SRS012969\_contig\_number\_13574SRS013098\_contig\_number\_20012SRS013687\_contig\_number\_8378SRS013800\_contig\_number\_2836SRS013951\_contig\_number\_37514SRS014235\_contig\_number\_33410SRS014287\_contig\_number\_16319SRS014412\_contig\_number\_contig-100\_912.119604SRS014683\_contig\_number\_4785SRS015264\_contig\_number\_3701SRS015579\_contig\_number\_18504SRS015694\_contig\_number\_14100SRS015782\_contig\_number\_27429SRS016018\_contig\_number\_15412SRS016056\_contig\_number\_2593SRS016132\_contig\_number\_1976SRS016203\_contig\_number\_72SRS016335\_contig\_number\_36279SRS016753\_contig\_number\_7297SRS016840\_contig\_number\_96SRS016954\_contig\_number\_13800SRS017103\_contig\_number\_26505SRS017247\_contig\_number\_1875SRS017433\_contig\_number\_9236SRS018133\_contig\_number\_4562SRS018575\_contig\_number\_3026SRS018836\_contig\_number\_21254SRS018888\_contig\_number\_3708SRS018936\_contig\_number\_6609SRS019030\_contig\_number\_5348SRS019161\_contig\_number\_8271SRS019178\_contig\_number\_2959SRS019286\_contig\_number\_117SRS019381\_contig\_number\_12701SRS019397\_contig\_number\_1917SRS019693\_contig\_number\_7332SRS019808\_contig\_number\_18545SRS019910\_contig\_number\_15821SRS019968\_contig\_number\_10365SRS020233\_contig\_number\_39062SRS020271\_contig\_number\_contig-100\_1474.31858SRS022524\_contig\_number\_13919SRS022609\_contig\_number\_15786SRS023526\_contig\_number\_35568SRS023583\_contig\_number\_4830SRS023914\_contig\_number\_21254SRS024009\_contig\_number\_5570SRS024075\_contig\_number\_11873SRS024331\_contig\_number\_33497SRS024435\_contig\_number\_20585SRS042628\_contig\_number\_5771SRS043841\_contig\_number\_8890SRS044535\_contig\_number\_21258SRS045004\_contig\_number\_14623SRS045645\_contig\_number\_2461SRS045739\_contig\_number\_2594SRS045826\_contig\_number\_1074SRS046068\_contig\_number\_970SRS046369\_contig\_number\_10729SRS046717\_contig\_number\_8023SRS047014\_contig\_number\_contig-100\_5666.185866SRS047044\_contig\_number\_6341SRS047433\_contig\_number\_11572SRS048981\_contig\_number\_2457SRS049712\_contig\_number\_17880SRS049773\_contig\_number\_31144SRS049896\_contig\_number\_6228SRS050026\_contig\_number\_9434SRS050299\_contig\_number\_5622SRS050422\_contig\_number\_28781SRS050520\_contig\_number\_237SRS050941\_contig\_number\_19182SRS051031\_contig\_number\_23623SRS051610\_contig\_number\_10289SRS052697\_contig\_number\_33494SRS053356\_contig\_number\_46441SRS053398\_contig\_number\_10983SRS054059\_contig\_number\_3717SRS054352\_contig\_number\_6426SRS054905\_contig\_number\_22381SRS055966\_contig\_number\_850SRS055982\_contig\_number\_1960SRS056519\_contig\_number\_10138SRS058770\_contig\_number\_14413SRS062427\_contig\_number\_4317SRS062654\_contig\_number\_21317SRS063040\_contig\_number\_19726SRS063127\_contig\_number\_2205SRS063489\_contig\_number\_2190SRS063985\_contig\_number\_23505SRS064232\_contig\_number\_389SRS065176\_contig\_number\_12572SRS065397\_contig\_number\_11180SRS065504\_contig\_number\_31594SRS074670\_contig\_number\_4051SRS075341\_contig\_number\_7673SRS075773\_contig\_number\_contig-100\_293.230178SRS075878\_contig\_number\_12548SRS075984\_contig\_number\_6127SRS076876\_contig\_number\_122SRS076976\_contig\_number\_14855SRS077086\_contig\_number\_8347SRS077231\_contig\_number\_24430SRS077552\_contig\_number\_12983SRS077641\_contig\_number\_18591SRS077849\_contig\_number\_13067SRS078176\_contig\_number\_2915SRS078242\_contig\_number\_17090SRS078419\_contig\_number\_29224SRS078665\_contig\_number\_9970SRS098073\_contig\_number\_12450SRS098571\_contig\_number\_32898SRS098644\_contig\_number\_26474SRS098655\_contig\_number\_12537SRS098717\_contig\_number\_13494SRS101268\_contig\_number\_482SRS101433\_contig\_number\_2030SRS104036\_contig\_number\_6499SRS104084\_contig\_number\_12764SRS1041031\_contig\_number\_contig-100\_766.161280SRS1041039\_contig\_number\_20086SRS1041091\_contig\_number\_18829SRS1041092\_contig\_number\_10146SRS1041112\_contig\_number\_2566SRS1041118\_contig\_number\_3390SRS1041130\_contig\_number\_13SRS1041134\_contig\_number\_3647SRS1041136\_contig\_number\_18768SRS1041137\_contig\_number\_8364SRS1041138\_contig\_number\_9972SRS1041139\_contig\_number\_1782SRS1041140\_contig\_number\_21419SRS1041141\_contig\_number\_8301SRS104311\_contig\_number\_36875SRS104327\_contig\_number\_3817SRS104400\_contig\_number\_22963SRS104912\_contig\_number\_2309SRS105082\_contig\_number\_4794SRS105153\_contig\_number\_6233SRS1054716\_contig\_number\_10656SRS1055022\_contig\_number\_10718SRS140513\_contig\_number\_8632SRS142503\_contig\_number\_32546SRS142542\_contig\_number\_22998SRS142890\_contig\_number\_35317SRS142923\_contig\_number\_14149SRS143148\_contig\_number\_2577SRS143181\_contig\_number\_626SRS143523\_contig\_number\_9710SRS143780\_contig\_number\_18199SRS143876\_contig\_number\_20519SRS143991\_contig\_number\_21528SRS144297\_contig\_number\_11837SRS144506\_contig\_number\_36358SRS144537\_contig\_number\_20780SRS144603\_contig\_number\_12922SRS145308\_contig\_number\_60SRS146764\_contig\_number\_24139SRS146812\_contig\_number\_62336SRS146813\_contig\_number\_27907SRS147346\_contig\_number\_32403SRS147425\_contig\_number\_4798SRS147557\_contig\_number\_13085SRS147614\_contig\_number\_1571SRS147653\_contig\_number\_6130SRS147977\_contig\_number\_3363SRS148091\_contig\_number\_9528SRS148196\_contig\_number\_29885SRS148253\_contig\_number\_9288SRS148424\_contig\_number\_19835SRS148721\_contig\_number\_20226SRS148817\_contig\_number\_10198SRS149325\_contig\_number\_4027SRS893230\_contig\_number\_7036SRS893292\_contig\_number\_1928SRS893295\_contig\_number\_2621SRS893300\_contig\_number\_173SRS893327\_contig\_number\_9551SRS893358\_contig\_number\_9481SRS893378\_contig\_number\_19706SRS893382\_contig\_number\_1977SRS893383\_contig\_number\_27735
183
46503
species

1

SRS051882\_contig\_number\_10589
species
823

2

SRS052078\_contig\_number\_1193SRS148159\_contig\_number\_23754
species
1263091

1263094
species

SRS013476\_contig\_number\_11855SRS024132\_contig\_number\_37749SRS077294\_contig\_number\_11805SRS097920\_contig\_number\_16788SRS147271\_contig\_number\_37938
5

387661
species

SRS011084\_contig\_number\_9108SRS015960\_contig\_number\_12375SRS016018\_contig\_number\_16049SRS019601\_contig\_number\_24022SRS021484\_contig\_number\_19876SRS021948\_contig\_number\_11205SRS023715\_contig\_number\_16606SRS077127\_contig\_number\_16523SRS1041129\_contig\_number\_10388SRS1041145\_contig\_number\_26151SRS146812\_contig\_number\_62368
11

43
family
815

1
816

SRS011405\_contig\_number\_3869
43
genus

3

SRS045244\_contig\_number\_838SRS048870\_contig\_number\_1947SRS049446\_contig\_number\_3905
species
246787

2

SRS013940\_contig\_number\_3209SRS019445\_contig\_number\_23070
species
329854

species
821
6

SRS013521\_contig\_number\_6000SRS015663\_contig\_number\_12530SRS098061\_contig\_number\_4458SRS104636\_contig\_number\_1776SRS1055069\_contig\_number\_4375SRS144714\_contig\_number\_16043

species
46506
5

SRS050752\_contig\_number\_4664SRS064757\_contig\_number\_12021SRS077589\_contig\_number\_1478SRS143722\_contig\_number\_3953SRS149181\_contig\_number\_11527

1

SRS148319\_contig\_number\_4846
species
2292931


SRS014613\_contig\_number\_527SRS043768\_contig\_number\_3003SRS049995\_contig\_number\_13826SRS101376\_contig\_number\_16546SRS104311\_contig\_number\_30823SRS104975\_contig\_number\_19547
6
47678
species


SRS097889\_contig\_number\_3824
1
2292936
species


SRS049402\_contig\_number\_5481
1
2292955
species

species
818
5

SRS022137\_contig\_number\_11130SRS075021\_contig\_number\_1876SRS143372\_contig\_number\_6667SRS147139\_contig\_number\_10112SRS893170\_contig\_number\_1546

1

SRS042966\_contig\_number\_20010
species
626931

291644
species

SRS049959\_contig\_number\_27962
1

820
species

SRS024492\_contig\_number\_10319
1

species
28116
3

SRS017821\_contig\_number\_9789SRS050925\_contig\_number\_15213SRS149075\_contig\_number\_7059


SRS014948\_contig\_number\_10271SRS017859\_contig\_number\_5234SRS018049\_contig\_number\_515SRS019685\_contig\_number\_10004SRS063190\_contig\_number\_1824
5
1638780
species

species
674529
1

SRS075821\_contig\_number\_20107

1
171550
family

1
239759
genus

1

SRS076804\_contig\_number\_17182
species
1872444

1
family
171552

genus
577309
1

454155
species

SRS893252\_contig\_number\_3125
1

1239
phylum
2

186801
class
2

2
186802
order

family
541000
1

1
genus
1263

2293224
species

SRS017521\_contig\_number\_36366
1

1
186803
family

1
genus
841

1

SRS058070\_contig\_number\_21413
species
166486
